# Relative contributions of six lifestyle- and health-related exposures to epigenetic aging: the Coronary Artery Risk Development in Young Adults (CARDIA) Study

**DOI:** 10.1186/s13148-022-01304-9

**Published:** 2022-07-07

**Authors:** Kyeezu Kim, Yinan Zheng, Brian T. Joyce, Hongmei Jiang, Philip Greenland, David R. Jacobs, Kai Zhang, Lei Liu, Norrina B. Allen, John T. Wilkins, Sarah N. Forrester, Donald M. Lloyd-Jones, Lifang Hou

**Affiliations:** 1 Department of Preventive Medicine, Northwestern University Feinberg School of Medicine, 680 Lake Shore Drive, Suite 1400, Chicago, IL 60611 USA; 2 Department of Statistics, Northwestern University, Evanston, IL USA; 3 Division of Epidemiology and Community Health, University of Minnesota School of Public Health, Minneapolis, MN USA; 4 Department of Environmental Health Sciences, University at Albany, State University of New York, Rensselaer, NY USA; 5 Division of Biostatistics, Washington University in St. Louis, St. Louis, MO USA; 6 Department of Population and Quantitative Health Sciences, University of Massachusetts Medical School, Worcester, MA USA

**Keywords:** DNA methylation, Epigenetic aging, Accelerated epigenetic age, Lifestyle- and health-related components

## Abstract

**Background:**

DNA methylation-based GrimAge acceleration (GrimAA) is associated with a wide range of age-related health outcomes including cardiovascular disease. Since DNA methylation is modifiable by external and behavioral exposures, it is important to identify which of these exposures may have the strongest contributions to differences in GrimAA, to help guide potential intervention strategies. Here, we assessed the relative contributions of lifestyle- and health-related components, as well as their collective association, to GrimAA.

**Results:**

We included 744 participants (391 men and 353 women) from the Coronary Artery Risk Development in Young Adults (CARDIA) study with blood DNA methylation information at CARDIA Exam Year (Y) 20 (2005–2006, mean age 45.9 years). Six cumulative exposures by Y20 were included in the analysis: total packs of cigarettes, total alcohol consumption, education years, healthy diet score, sleep hours, and physical activity. We used quantile-based g-computation (QGC) and Bayesian kernel machine regression (BKMR) methods to assess the relative contribution of each exposure to a single overall association with GrimAA. We also assessed the collective association of the six components combined with GrimAA. Smoking showed the greatest positive contribution to GrimAA, accounting for 83.5% of overall positive associations of the six exposures with GrimAA (QGC weight = 0.835). The posterior inclusion probability (PIP) of smoking also achieved the highest score of 1.0 from BKMR analysis. Healthy diet and education years showed inverse contributions to GrimAA. We observed a U-shaped pattern in the contribution of alcohol consumption to GrimAA. While smoking was the greatest contributor across sex and race subgroups, the relative contributions of other components varied by subgroups.

**Conclusions:**

Smoking, alcohol consumption, and education showed the highest contributions to GrimAA in our study. Higher amounts of smoking and alcohol consumption were likely to contribute to greater GrimAA, whereas achieved education was likely to contribute to lower GrimAA. Identifying pertinent lifestyle- and health-related exposures in a context of collective components can provide direction for intervention strategies and suggests which components should be the primary focus for promoting younger GrimAA.

**Supplementary Information:**

The online version contains supplementary material available at 10.1186/s13148-022-01304-9.

## Introduction

Lifestyle- and other health-related exposures such as smoking, alcohol consumption, education, diet, and physical activity are associated with age-related health outcomes including all-cause mortality [1], cancer [2–4], and cardiovascular disease (CVD) [5–7]. Lifestyle- and health-related exposures can also trigger epigenetic modifications, which in turn affect expression of genes related to age-related health outcomes [8]. Growing evidence suggests that components such as smoking, alcohol consumption, education, and physical activity are associated with accelerated- or decelerated epigenetic age [9–11]. Epigenetic age, an alternative to chronological age, was proposed and developed to overcome the limitation of use of chronological age due to inter-individual variability in age-related biological changes as well as health-related exposures [12, 13].

Epigenetic marks represented by DNA methylation accumulate with lifestyle and environmental exposures over time [8, 14]; thus DNA methylation-based biological age (epigenetic age) has been suggested as a useful biomarker of age-related conditions [12, 15]. Several epigenetic age estimates have been proposed to date, including extrinsic and intrinsic epigenetic age acceleration (EEAA and IEAA, respectively), and more recently, Levine et al.’s PhenoAge and Lu et al.’s GrimAge [16–19]. Typically, these epigenetic age estimates represent biological/epigenetic age as a summary measurement based on numbers of age-related CpGs (353 CpGs for IEAA, 71 for EEAA, 513 for PhenoAge, and 1030 for GrimAge, respectively) [16–19]. Prior studies showed that accelerated epigenetic age (older epigenetic age than chronological age) is associated with age-related health outcomes such as diabetes, metabolic syndrome, cancer incidence and mortality, CVD, and all-cause mortality [19–23]. Studies also suggested that PhenoAge acceleration (PhenoAA) and GrimAge acceleration (GrimAA) are more powerful than older epigenetic aging estimators in assessing associations with health outcomes [19, 24]; however, the number of studies with associations of upstream lifestyle- and health-related exposures during young adulthood with PhenoAA and GrimAA measured in middle age are still limited [25]. Therefore, the extent to which epigenetic aging estimators capture information on prior lifestyle and other health-related risk factors remains incompletely understood.

Since many lifestyle- and health-related exposures correlate with one another [11], assessing multiple components as a combination is important because of potential synergistic interactions [1], particularly when studying chronic disease risks [1, 26]. Most studies of lifestyle and epigenetic age acceleration (EAA) have focused on single lifestyle- or health-related behaviors or exposures (e.g., smoking, alcohol consumption, education, etc.) and evaluated the association with EAA using traditional regression models, assuming independence among the components and additivity of effects on the outcome [9, 10], which may lead to confounded associations. EAA is a reflection of multiple factors comprising various lifestyle and environmental exposures, and the joint effect of multiple components on EAA may be greater or lesser than the sum of a single exposure’s effect. Furthermore, it is challenging to identify impactful exposures to EAA for public health intervention strategies without considering the coexistence of other components with complex relationships. To overcome these challenges, we adapt two advanced statistical approaches: quantile-based g-computation (QGC) [27] and Bayesian kernel machine regression (BKMR) [28]. Designed to deal with multiple exposures simultaneously, the strengths of the two methods include identification of relative contributions for each exposure to the collective association with the outcome [27, 28].

In this study, we aimed to investigate the collective association and relative contribution of lifestyle- and health-related exposures with epigenetic aging biomarkers. We aimed to assess six established exposures and health behaviors as components in a model of their relative contributions to the collective association with EAA. Using a subpopulation with DNA methylation information from the Coronary Artery Risk Development in Young Adults (CARDIA) study, we examined the collective association of the combination of lifestyle- and health-related components with EAA (represented by PhenoAA and GrimAA), as well as the relative contributions of individual components to the collective association.

## Results

### Distribution of participants’ characteristics

**Fig. 1:**
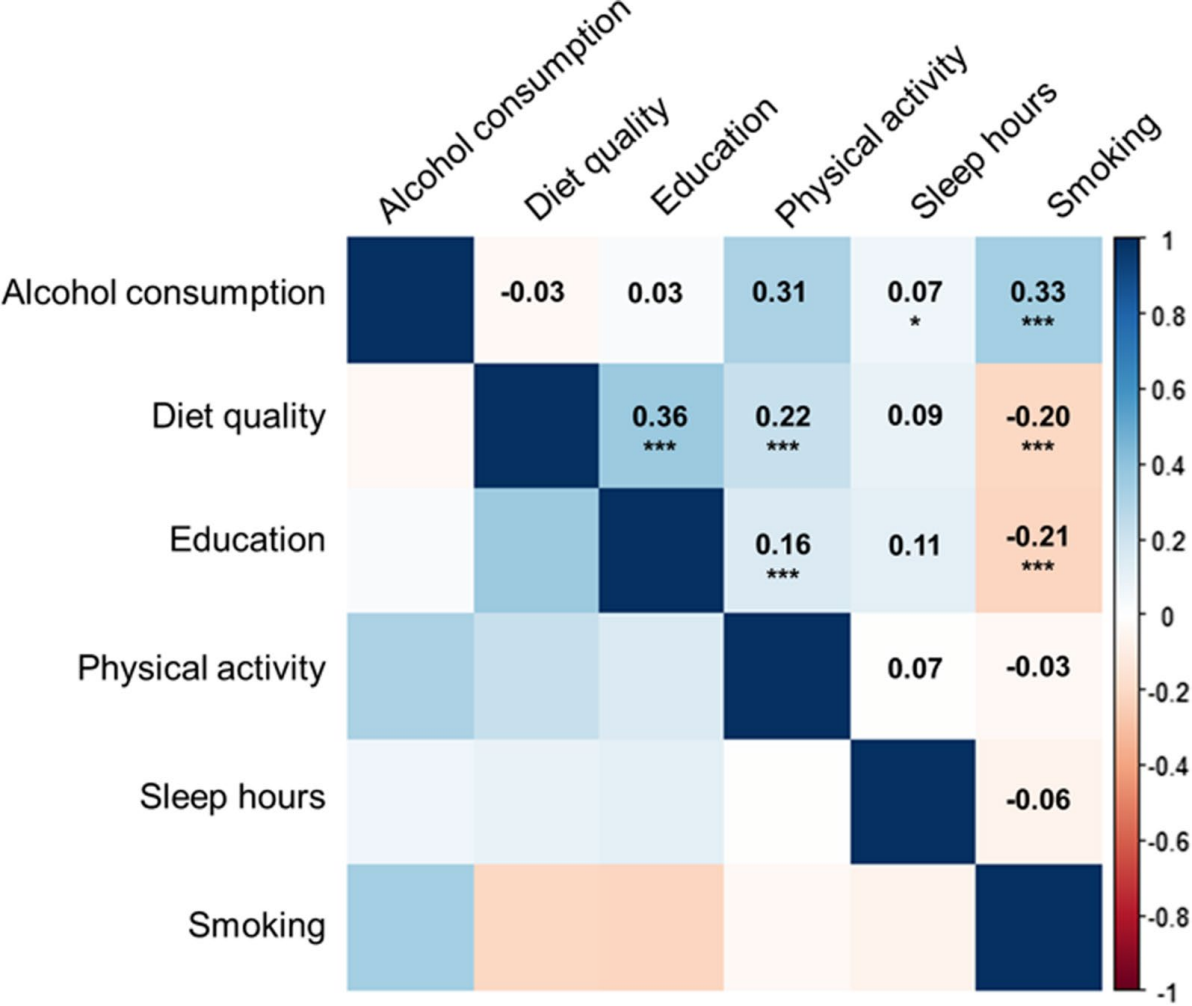
Spearman correlations among six cumulative lifestyle- and health-related components

Table 1 represents the distribution of study participants' characteristics. A total of 744 participants (391 men and 353 women) were included in the study, consisting of 304 Black participants and 440 White participants. The mean age of study participants was 45.9 (standard deviation [SD]: 3.5) years. Figure 1 displays Spearman correlations among the six lifestyle- and health-related exposures. The strongest correlation was shown between diet and education (R: 0.36), followed by the correlation between smoking and alcohol consumption (R: 0.33). The correlations by race- and sex subgroups were generally consistent with the results from all participants (Additional file 1: Fig. S1).

**Table 1:** Distributions of six cumulative lifestyle- and health-related components among study participants from CARDIA study

Variables	Description	Mean (SD)	IQR
Age	Chronological age, at Y20	45.9 (3.5)	5.7
Sex, N (%)	Men	391 (52.6)	
	Women	353 (47.5)	
Race, N (%)	Black participants	304 (40.9)	
	White participants	440 (59.1)	
Smoking	Total packs of cigarettes by Y20 (for former and current smokers)	1717.5 (3348.4)	1764.0
Smoking status at Y20, N (%)	Never	459 (61.7)	
	Former	147 (19.8)	
	Current	138 (18.6)	
Alcohol consumption	Total grams of alcohol consumption by Y20	227.5 (341.6)	298.0
Diet	Average Healthy Eating Index score, over Y0, Y7, and Y20	67.6 (11.8)	15.9
Education	Total years of education, at Y20	15.1 (2.5)	3.0
Physical activity	Cumulative total intensity score by Y20	350.9 (279.0)	340.0
Sleep hours	Average sleeping hours in the past 30 days, at Y15 and Y20	6.7 (1.3)	1.3
BMI	Body mass index (kg/m^2^), at Y20	29.3 (6.4)	7.5
Field center, N (%)	Birmingham	197 (26.5)	
	Chicago	174 (23.4)	
	Minnesota	181 (24.3)	
	Oakland	192 (25.8)	

*SD* standard deviation, *IQR* interquartile range

#### Relative contribution of six exposures and collective association

**Fig. 2:**
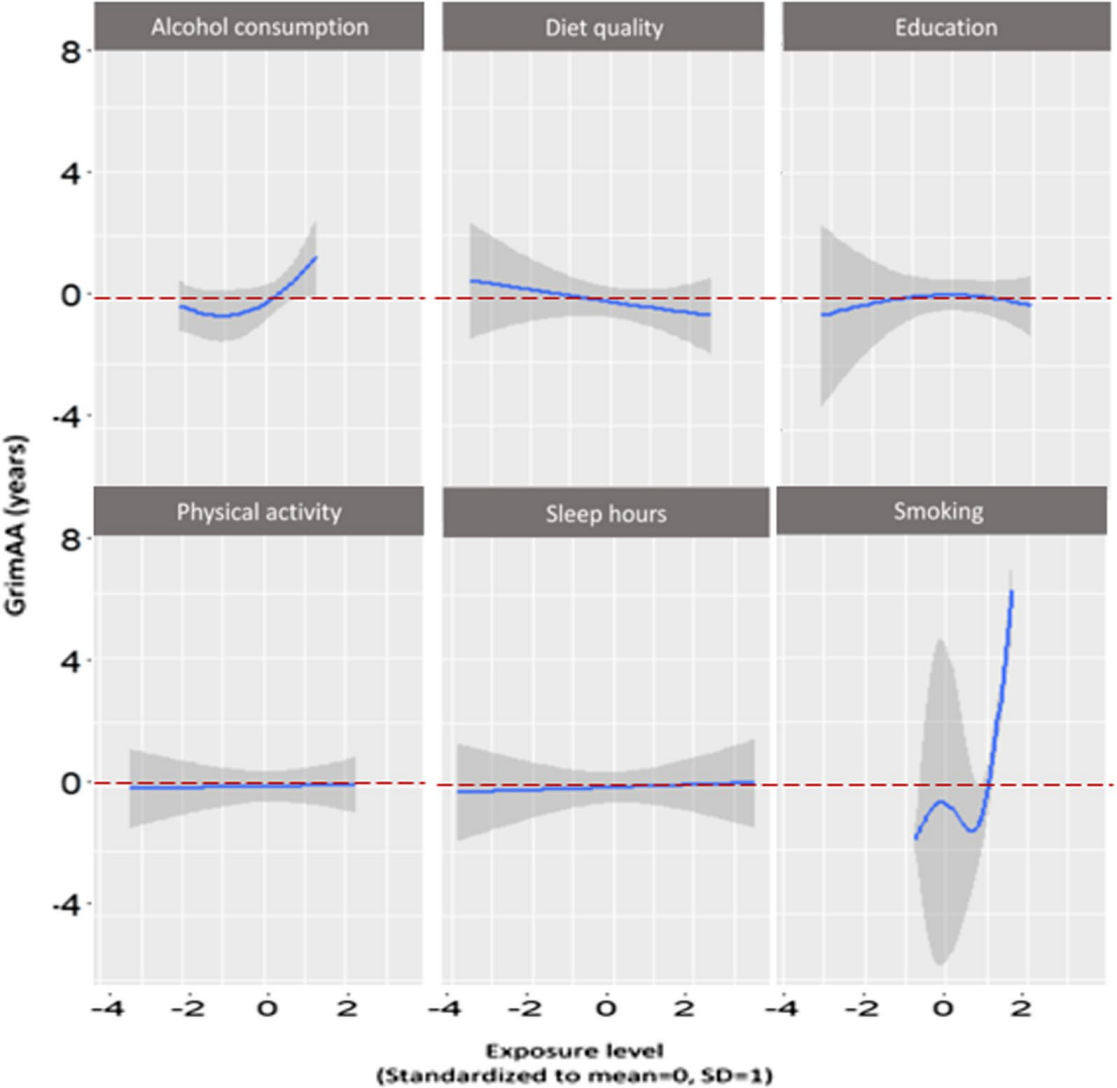
The exposure–response pattern (blue lines) with 95% confidence intervals (gray shade) for each lifestyle- and health-related exposure) with other exposures fixed at median). The *X*-axis represents standardized levels (mean = 0, standard deviation [SD] = 1) of six lifestyle- and health-related exposures. The *Y*-axis represents GrimAA, in years. A GrimAA of zero (dotted red line) indicates an epigenetic age equal to chronological age. GrimAA less than zero (below the dotted red line) indicates younger epigenetic age compared to chronological age

Table 2 shows the relative contribution of each exposure, and the collective association of all six components, to GrimAA. For the collective association between the six components and GrimAA using QGC, we observed an average of 5.15 years greater GrimAA per quartile difference in the six components (beta: 5.15, 95% CI 4.16, 5.86). In QGC, smoking showed the greatest relative contribution to greater GrimAA, accounting for 83.5% of contribution to the positive association (weight: 0.835). Alcohol consumption (weight: 0.129), sleep hours (weight: 0.010), and physical activity (weight: 0.024) also contributed to the positive association with GrimAA, whereas diet quality (weight: − 0.537) and education (weight: − 0.463) contributed to an inverse (and favorable) association with GrimAA. The estimate of collective association from BKMR was 3.62 years, and the 95% credible interval (CrI) did not include the null (95% CrI 1.93, 5.22). Using BKMR, we observed that smoking showed the greatest contribution with highest PIP (PIP: 1.000), followed by alcohol consumption (PIP: 0.997), education (PIP: 0.812), diet quality (PIP: 0.310), sleep hours (PIP: 0.091), and physical activity (PIP: 0.040) in GrimAA. Figure 2 represents the patterns of single exposure associations with GrimAA from BKMR analysis among the total population. We observed a U-shaped pattern with alcohol consumption and GrimAA. The patterns with other components were generally consistent with the direction of associations shown in QGC analysis. Diet quality showed a linear and negative association with GrimAA. Education showed an overall negative association with GrimAA. Smoking showed non-monotonic response functions, but the highest levels of smoking displayed a positive association with GrimAA. The patterns for physical activity and sleep hours were plateaus, consistent to the minimal PIPs of the two components in Table 2.

**Table 2:** Relative contributions of six components to GrimAA in the CARDIA sample from QGC and BKMR

Lifestyle components by Y20	Weights from QGC	PIPs from BKMR
Alcohol consumption	0.129	0.997
Diet quality	− 0.537	0.310
Education years	− 0.463	0.812
Physical activity	0.024	0.040
Sleep hours	0.010	0.091
Smoking	0.835	1.000
*Collective association†*	5.15 (95% CI 4.16, 5.86), *p* < 0.001	3.62 (95% CrI 1.93, 5.22)

Models were adjusted for race, sex, body mass index (BMI), and field center; *CI* confidence interval, *CrI* credible interval, *QGC* quantile-based g-computation, *BKMR* Bayesian kernel machine regression, *PIPs* posterior inclusion probabilities. The positive and negative weights from QGC represents the proportion of the effect estimate for each component (sum up to 1 or − 1 for the same direction); The PIP reflects the ranked importance of each component in association with GrimAA. *†* Change in mean GrimAA per one quartile change of all six components for QGC; change in mean GrimAA when all of the six lifestyle components are fixed at their 75th percentile compared to when the six lifestyle components are at their 25th percentile for BKMR

The results from PhenoAA among total participants are presented in Additional file 1: Table S1. The average collective association between the collective components and PhenoAA from QGC was 1.64 years (95% CI 0.15, 3.13), per quartile difference in the six components. Using QGC, we observed that smoking (weight: 0.861) and diet quality (weight: − 0.690) showed the greatest positive and negative weights, respectively. The collective association from BKMR was 1.30 years; however, the 95% CrI included the null (95% CrI − 0.89, 3.49). In BKMR, smoking showed the highest PIP of 0.955.

#### Results from the stratified analysis by study subgroups

Table 3 shows the results from the subgroup-specific analyses, stratified by participants’ smoking status at Y20, sex, and race, respectively.

**Table 3:** Relative contributions of six components to GrimAA at Y20 from QGC and BKMR by subgroups

Lifestyle components at or by Y20	Weights from QGC		PIPs from BKMR	
By smoking status	Ever smokers (*N* = 285)	Never smokers (*N* = 459)	Ever smokers (*N* = 285)	Never smokers (*N* = 459)
Alcohol consumption	0.361	0.405	0.868	0.429
Diet quality	− 0.445	− 0.762	0.471	0.203
Education years	− 0.555	− 0.238	0.676	0.086
Physical activity	0.071	0.325	0.135	0.142
Sleep hours	0.007	0.270	0.138	0.119
Smoking	0.559	NA	1.000	NA
*Collective association*	1.47 (95% CI 0.39, 2.55), *p* = 0.008	0.13 (95% CI − 0.43, 0.71), *p* = 0.639	3.08 (95% CrI 1.13, 5.02)	0.47 (95% CrI − 0.38, 1.32)

By sex	Men (*N* = 391)	Women (*N* = 353)	Men (*N* = 391)	Women (*N* = 353)
Alcohol consumption	0.136	0.093	0.995	0.401
Diet quality	− 0.224	− 0.692	0.012	0.617
Education years	− 0.776	− 0.267	0.061	0.193
Physical activity	0.032	0.005	0.109	0.016
Sleep hours	0.034	− 0.036	0.053	0.055
Smoking	0.798	0.907	1.000	1.000
*Collective association*	6.67 (95% CI 5.43, 7.91), *p* < 0.001	3.18 (95% CI 1.92, 4.44), *p* < 0.001	5.53 (95% CrI 3.78, 7.27)	2.52 (95% CrI 0.74, 4.28)

By race	Black participants (*N* = 304)	White participants (*N* = 440)	Black participants (*N* = 304)	White participants (*N* = 440)
Alcohol consumption	0.168	0.082	0.993	0.292
Diet quality	− 0.477	− 0.423	0.032	0.425
Education years	0.034	− 0.577	0.063	0.821
Physical activity	− 0.523	0.061	0.001	0.410
Sleep hours	0.018	0.032	0.028	0.217
Smoking	0.780	0.824	1.000	1.000
*Collective association*	6.03 (95% CI 4.45, 7.61), *p* < 0.001	4.52 (95% CI 3.46, 5.58), *p* < 0.001	5.93 (95% CrI 3.24, 8.61)	1.96 (95% CrI 0.11, 3.81)

Models were adjusted for race, sex, body mass index (BMI), and field center; *CI* confidence interval, *CrI* credible interval, *QGC* Quantile-based g-computation, *BKMR* Bayesian kernel machine regression, *PIPs* posterior inclusion probabilities. The positive and negative weights from QGC represents the proportion of the effect estimate for each component (sum up to 1 or − 1 for the same direction); The PIP reflects the ranked importance of each component in association with GrimAA. *†* Change in mean GrimAA per one quartile change of all six components for QGC; change in mean GrimAA when all of the six lifestyle components are fixed at their 75th percentile compared to when the six lifestyle components are at their 25th percentile for BKMR

*Results by smoking status:* In QGC analysis, the collective association of the six components with GrimAA from QGC among ever smokers was 1.47 (95% CI 0.39, 2.55). Smoking showed the greatest contribution (55.9%) to the positive association with GrimAA with the highest positive weight (weight: 0.559) among ever smokers. Education showed the greatest contribution (55.5%) to the inverse association with GrimAA (weight: − 0.555), followed by diet quality (weight: − 0.445) among ever smokers. Using BKMR, the collective association of the six components with GrimAA was 3.08 years, and the 95% credible interval (CrI) did not include the null (95% CrI 1.13, 5.02). In BKMR among ever smokers, smoking showed the highest PIP (PIP: 1.000), followed by alcohol consumption (PIP: 0.868), education (PIP: 0.676), and diet quality (PIP: 0.471). The collective associations using QGC and BKMR presented null associations among never smokers. Among never smokers, alcohol consumption showed the greatest contribution (40.5%) to the positive association with GrimAA with a weight of 0.405. Diet quality showed the greatest negative weight (weight: − 0.762) among never smokers. In BKMR, the PIP for the five other components in never smokers were relatively low, ranging from 0.086 to 0.429. In the separate analyses by grouping participants into current, former, and never smokers, the relative contributions of components between current and former smokers were generally consistent (data not shown).

*Results by sex:* In the analysis stratified by sex (Table 3), we observed positive collective associations between the six components and GrimAA using QGC in both men and women, as average 6.67 years higher GrimAA per quartile difference (95% CI 5.43, 7.91) for men and 3.18 years higher GrimAA per quartile change (95% CI 1.92, 4.44) for women, respectively. Smoking showed the greatest contribution to the positive association with GrimAA in both sexes (weights: 0.798 for men and 0.907 for women, respectively) using QGC. In men, education years showed the greatest contribution to the inverse association with GrimAA (weight: − 0.776). In women, diet quality showed the greatest negative contribution to the association with GrimAA (weight: − 0.692). In BKMR, the collective associations between the collective lifestyle and GrimAA using BKMR did not include the null in either sex stratum, with an average increase of 5.53 years in GrimAA (95% CrI 3.78, 7.27) for men and 2.52 years (95% CrI 0.74, 4.28) for women, respectively. Smoking showed the highest PIP for GrimAA in both sexes (PIP: 1.00 for both men and women). In men, alcohol consumption also showed a high PIP of 0.995. In women, diet quality (PIP: 0.617), and alcohol consumption (PIP: 0.401) followed the PIP of smoking. We also observed smoking as a strong contributor to the positive association with PhenoAA in both sexes (Additional file 1: Table S2).

*Results by race:* In the analysis stratified by race, smoking again showed high weights and PIPs for GrimAA (Table 3). The collective association using QGC showed an average of 6.03 years greater GrimAA per quartile difference in the six components (95% CI 4.45, 7.61) in Black participants and 4.52 years (95% CI 3.46, 5.58) in White participants, respectively. Using QGC, we observed smoking accounted for 78.0% of positive association with GrimAA in Black participants (weight: 0.780) and 82.4% in White participants (weight: 0.824), respectively. Education showed a strong inverse contribution (weight: − 0.577) in White participants only. Diet quality showed negative contributions for both Black and White participants (weight: − 0.477 for Black participants and − 0.423 for White participants, respectively). The collective associations using BKMR were 5.93 (95% CrI 3.24, 8.61) for Black participants and 1.96 (95% CrI 0.11, 3.81) for White participants, respectively, and both did not include the null. Using BKMR, we observed a PIP of 1.000 for smoking in both races. Alcohol consumption showed a PIP of 0.993 in Black participants. Education showed a PIP of 0.821 in White participants. We also observed smoking showed high weights and PIPs for PhenoAA in both races using both QGC and BKMR (Additional file 1: Table S2).

The six components in linear regression models had associations that were generally consistent with the results from QGC and BKMR (Additional file 1: Table S3). We did not observe differences using moderate or vigorous levels of physical activity instead of total intensity score (data not shown).

## Discussion

In this study, we examined six lifestyle- and health-related exposures and their individual and combined association with EAA and explored their relative contributions to EAA. Among the individual components, we observed smoking and alcohol consumption, diet quality, and education had significant contributions to EAA. Smoking showed the greatest contributions in all analyses. We also observed lifestyle- and health-related exposures that showed stronger contributions in subgroup-specific analyses, such as diet quality among women and education among White participants. Sleep hours and physical activity did not show strong contributions to the collective association between the six components and greater EAA.

Our results support prior findings on smoking and EAA [29, 30] and suggest that the influence of smoking may overwhelm that of other lifestyle- and health-related components in our study. Of note, the DNA methylation surrogate marker for smoking pack-years was used in defining GrimAge. However, smoking also showed the strongest contribution to PhenoAA, which does not include smoking pack-years in its calculation. The strong contribution of smoking to both EAA measurements implies that greater cumulative smoking plays a role in epigenetic changes captured by EAA, once again highlighting the primacy of smoking exposure in the risk of age-related diseases. These findings reinforce the need for smoking cessation efforts, and may help enhance them; future studies should confirm our findings in additional populations and explore the possibility of including epigenetic components in anti-smoking interventions.

Using BKMR, we observed a U-shaped relationship between alcohol consumption and GrimAA similar to the observed nonlinear relationship between alcohol consumption and CVD risk [31]. Together with studies associating alcohol consumption with DNA methylation surrogate markers such as C-reactive protein, leptin, and PAI-1 [32–34] this evidence suggests that epigenetic changes represented by GrimAA may be in the pathway between alcohol consumption and health outcomes including CVD. Furthermore, GrimAA may serve as a tool to reflect the amounts of alcohol consumption for appropriate intervention, rather than self-reported values. Identifying the acting mechanism and causality between alcohol consumption and GrimAA would be worth investigating in future studies. Similarly, the results of diet quality and EAA in our study are in-line with previous evidence, which indicates a healthier diet is associated to decelerated epigenetic aging [35, 36]. Prior studies reported that higher education attainment was associated with slower EAA [10, 11, 30]. As a proxy of socioeconomic status, education level can connote various other factors connected to health such as financial status/income, employment, and access to higher-quality food [37]. The possibility that these factors contribute most to GrimAA, rather than education, should be explored in future studies.

In our stratified analyses by smoking status, lifestyle- and health-related components were not associated with GrimAA among never-smokers in combination. However, previous studies reported the association between GrimAA and adverse health outcomes such as metabolic syndrome and major depressive disorder among non-smokers [38, 39]. Our sex-stratified analysis using BKMR presented strong contributions of diet quality to GrimAA in women only, possibly reflecting different diet patterns by gender [40–42] which are not captured by a summary measurement such as HEI score. We also observed a contrast in the contributions of alcohol consumption and education between Black and White participants. Our findings of racial discrepancies in contributions of alcohol and education to GrimAA imply that those health-related components could have different associations with epigenetic changes by race, or racial disparities in lifestyle and environment. Other components associated with health disparities in sex and race (such as neighborhood environment, behavioral- and psychosocial factors) might play a role in the discrepancies by subgroups, would be worth being investigated, and should be researched carefully as these factors (such as education) could play a role as a proxy of other socioeconomic disparities. We believe identifying these different susceptibilities by demographic and behavioral subgroups should be a direction for future studies, which would provide important keys for targeted interventions for health promotion.

We observed modest discrepancies between the results for relative contributions from the QGC and BKMR analyses. One potential reason is differences in the measurements of ‘relative contribution’ between QGC and BKMR, captured by weight and PIP, respectively. While the weight in QGC reflects the proportion of effect due to certain components among all components in the same direction, the PIP in BKMR reflects the ranked importance of certain components in association with the outcome. As the relative contribution is a form of proportion in QGC, one component can have a low weight due to a competing component with a stronger contribution, yet still make a contribution to the collective association that might not be captured in QGC. On the other hand, a component with high weights may only indicate that that particular component has the greatest contribution compared to the others with the same direction of association, not necessarily a large and clinically meaningful contribution to the outcome on its own. Therefore, examining PIPs from BKMR alongside weights from QGC provides more information for understanding the relative contributions of whole components than either method singly. Another possible explanation for differences between the two approaches taken here may be the robustness and flexibility of the BKMR method [28]. Although QGC demonstrates the directions of association and relative contribution of single components by that direction, BKMR may reflect more accurate patterns of association such as nonadditive and nonlinear relationships. The case of alcohol consumption, which we found to have a U-shaped pattern with GrimAA, could be an example for this explanation as it showed low weights in most QGC analysis but high PIPs in BKMR. This shows how the parallel use of both methods is useful to understand the exposure-outcome relationship, comprising overall collective directions as well as potential nonlinearity of each component.

Our study has some limitations. We only focused on quantitative aspects of lifestyle- and health-related exposures in this study; however, qualitative measurements for those components can also affect EAA. For example, quality of sleep such as irregular sleep or sleep-disordered breathing may have greater contributions to EAA rather than sleep duration. For physical activity, different types of exercise (i.e., aerobics, weight-lifting) may have different impacts on EAA which are not captured by intensity score. Developing a way to incorporate qualitative measurements of lifestyle- and health-related components and identifying attributable components for EAA could be one direction for future studies. Second, there is no established standard to construct a measure of 'collective lifestyle- and health-related exposure'. Other components including occupation or environmental exposure, which were not focused on in the current study, may have greater influence on EAA. Thus, it should be noted that residual confounding from components that were not measured in the current study may exist. Identifying impactful components using a more comprehensive set of lifestyle- and health-related components should also be a future direction of the studies with lifestyle- and health-related components and EAA. In addition, as our study focused on the lifestyle- and health-related exposures that are modifiable by public health promotions such as behavioral interventions or health policy, the acting mechanisms through clinical determinants (e.g., blood glucose, lipid metabolism, etc.) of the combined exposures on EAA remained as a challenge. It would be worthwhile to be investigated in future research on the role of lifestyle- and health-related exposures and EAA. Finally, the proportions of current- and former smokers in our study are relatively small; however, they are comparable to the national samples in the USA [43], and we thus believe the data for this study provide external validity.

## Conclusions

EAA itself is a multifactorial trait. Lifestyle, behavioral, and environmental exposures influence EAA by altering DNA methylation levels. The positive overall association of lifestyle- and health-related components and GrimAA in our study supports the idea that multiple lifestyle- and health-related components collectively play a role in EAA. By identifying the relative contributions of lifestyle- and health-related exposures, we anticipate our study can provide a direction for intervention strategies, suggesting which components should be the primary focus for promoting younger EAA. Differences by race and sex from our results can also provide a key for targeted intervention strategies, with different primary focus by population.

## Methods

### Study samples

The study participants were from the Coronary Artery Risk Development in Young Adults (CARDIA) study. The CARDIA study is a multicenter prospective cohort study to examine the development and determinants of cardiovascular disease in young adults. At study baseline (Year 0; 1985–1986) 5115 Black and White adults (based on participants’ self-reported race) aged 18–30 years were enrolled across four field centers in Birmingham, AL, Chicago, IL, Minneapolis, MN, and Kaiser Permanente Health Plan in Oakland, CA. Participants have been followed up after baseline nine times, in study years (Y) Y0, Y2, Y5, Y7, Y10, Y15, Y20, Y25, and Y30. More details about study design and recruitment for the CARDIA study are described elsewhere [44]. In the current study, we included participants with complete information on DNA methylation and six lifestyle- and health-related exposures measured at Y20: alcohol consumption, education, diet, physical activity, sleep, and smoking. Of 3549 participants in CARDIA Y20, 1200 with available whole blood samples were randomly selected for DNA methylation measurement. After excluding samples with low DNA amount or poor quality, DNA methylation levels were measured in 1092 participants, and the final methylation dataset included 957 participants after quality control (QC) procedures. Out of 957 participants, we included participants with complete information across six component variables, which resulted in the final analytic data set of 744 participants.

### Lifestyle- and health-related components in CARDIA study

In the current study, we investigated six lifestyle- and health-related exposures: smoking, alcohol consumption, education, sleep, diet, and physical activity (as of Y20). Smoking was defined as cumulative packs of cigarettes by Y20, which was calculated as the total number of cigarette packs over the study years. For example, smoking from Y15 to Y20 was calculated by summing the number of self-reported daily amount of cigarettes at Y15 and Y20 multiplied by 5, and then divided by 20 (number of cigarettes in a pack). We repeated and summed this calculation for each exam interval. We defined alcohol consumption by Y20 as the summed average alcohol consumption for each pair of consecutive examinations multiplied by the time interval (in years) between them. For example, alcohol consumption from Y15 to Y20 was calculated by taking the mean of self-reported alcohol intakes (in ml/day) at Y15 and Y20 multiplied by 5. We repeated this calculation for each interval (Y0-Y2, Y2-Y5, Y5-Y7, Y7-Y10, Y10-Y15, Y15-Y20) and summed them together to produce a measure of alcohol consumption from Y0 to Y20. For education we used self-reported education (maximum education years) in years at Y20. Sleep information was obtained from the *Sleep Habits* questionnaire at Y15 and Y20, asking study participants to report the hours of actual sleep during the past month of the exam. We used the mean value of sleep hours between Y15 and Y20 for the analysis. The Healthy Eating Index (HEI) scores [45] were measured at Y0, Y7, and Y20 as a measure of participants' diet quality, asking participants’ self-reported dietary history. To calculate diet quality over the study years, we used mean values of HEI scores according to participants’ number of exam years [46]. Finally, for participants' physical activity levels, total intensity scores obtained from the self-reported *Physical Activity* questionnaire at Y0, Y2, Y5, Y7, Y10, Y15, and Y20 were employed in the current study. To calculate cumulative total intensity scores, we summed average total intensity scores for each pair of consecutive examinations multiplied by the time interval, as we adopted the same approach for alcohol consumption.

### Quality control of DNA methylation profiling and calculation of epigenetic age acceleration (EAA)

We conducted QC procedures adopting the R packages *minfi* [47] and *ENmix* [48] with DNA methylation profiles among 1200 participants. We excluded CpGs with detection rate less than 95%. We also excluded samples if the sample demonstrated either > 5% of low quality of methylation measurements or < 3 standard deviation from the mean intensity of bisulfite conversion probes. We further adopted Tukey's method to detect and exclude outlier samples [49]. The R function, *preprocessIllumina*, in the *minfi* package was used for preprocessing procedures after QC [47].

We used two EAA measurements, GrimAA and PhenoAA, to assess the association between collective lifestyle- and health-related components and epigenetic aging in the current study. We focused on GrimAA and PhenoAA because the two EAA measurements, which are recently developed, have shown better performance in association with health outcomes compared to the first-generation EAA measurements, as they were designed to predict healthspan [18, 19, 24]. The calculations of GrimAge and PhenoAge in participants were based on the published algorithms [18, 19]. We used Horvath's online DNA Methylation Age Calculator (https://dnamage.genetics.ucla.edu) to calculate GrimAge and PhenoAge with participants' DNA methylation data from the CARDIA study. GrimAA and PhenoAA were then derived from the regression residuals of GrimAge against chronological age, which captures the difference between epigenetic age and chronological age.

### Statistical analysis

We performed descriptive analyses to explore the distribution of participants' chronological age, GrimAge, and GrimAA as well as their lifestyle- and health-related exposures and potential confounders (body mass index [BMI, kg/m^2^], sex, race, and field center). The six components were log-transformed and centered and each rescaled to have a mean of 0 and a standard deviation (SD) of 1. We also assessed Spearman correlation coefficients among our six individual components of interest. To assess the relative contributions and collective associations of the six exposures, we used quantile-based g-computation (QGC) and Bayesian kernel machine regression (BKMR). For comparison purposes we conducted additional, conventional analyses with linear regression models by including all six components as independent variables and each EAA as an outcome. To be consistent with QGC and BKMR analyses, log-transformed and standardized (mean = 0, SD = 1) components variables were used in the linear regression models adjusting for the same covariates as the other two approaches. The slope coefficients from the linear models was defined as mean change of EAA per increase of 1 SD. Statistical significance was defined with a threshold of *p* value < 0.05.

We used SAS version 9.4 (SAS Institute Inc., Cary, NC) for descriptive analyses and traditional regression models, and R version 4.0.3 (R Core Team, 2020) for QGC and BKMR analyses. As we observed greater associations with GrimAA, we present GrimAA in our main results, and PhenoAA in our supplementary tables for all analyses.

#### Quantile-based g-computation (QGC)

In QGC, each continuous component is transformed into the quantized version, ${\text{Component}}_{j}^{q}$ (coded as 0, 1, 2, or 3), and fitted to a linear model as follows:

$$Y_{{{\text{EAA}}}} = \beta_{0} + \psi \mathop \sum \limits_{j = 1}^{6} w_{j} {\text{Component}}_{j}^{q} + \epsilon_{i} = \beta_{0} + \mathop \sum \limits_{j = 1}^{6} \beta_{j} {\text{Component}}_{j}^{q} + \epsilon_{i}$$

where *β*_*j*_ is the effect size for *j*th component, and $\epsilon_{i}$ is the error term. The estimate of collective association, $\psi = \sum\nolimits_{j = 1}^{6} {\beta_{j} }$, is interpreted as the change in EAA per one quartile change of all six components, controlling for covariates (age, sex, race, BMI, and field center). The weight for the *k*th component is defined as $w_{k} = \beta_{k} /\sum\nolimits_{j = 1}^{6} {\beta_{j} }$, when the directions are same across all components. If the directions are different, the weights are defined for each direction, thus sum to 1.0 for positive and to − 1.0 for negative. The R package *qgcomp* [27] was used to obtain point estimates and 95% confidence intervals (95% CI) for QGC analyses.

#### Bayesian kernel machine regression (BKMR)

We also used BKMR with a Gaussian kernel to investigate the association between the collective effect of six components and EAA. The equation of the BKMR model for this study can be represented as follows:

$$Y_{{{\text{EAA}}}} = h\left( {{\text{Component}}_{i} } \right) + {\text{Covariate}}_{i}^{{\text{T}}} \beta + \epsilon_{i}$$

where ${\text{Component}}_{i} = \left( {{\text{Component}}_{i1} , \ldots ,{\text{Component}}_{i6} } \right)$ is a vector of the six component variables for the *i*th participant, ${\text{Covariate}}_{i}^{{\text{T}}}$ is the matrix of covariates (age, sex, race, BMI, and field center), *β* is the vector of corresponding coefficients for covariates, and $\epsilon_{i}$ is the error term. In the context of this study, *h*() represents the unknown exposure–response relationship among the components in the combination, which may incorporate nonlinearity and nonadditivity. In this study, single exposure–response relationship was defined between each component and EAA, fixing all other components to their median and controlling other covariates. In BKMR, a kernel machine is used to specify the unknown exposure-relationship *h*(), and the component-wise variable selection using Gaussian kernel within a Bayesian paradigm allows to calculate posterior inclusion probability (PIP) [28]. The estimates for collective association were defined as the change in the mean EAA when all of the six components are fixed at their 75th percentile compared to when the six components are at their 25th percentile, controlling for covariates [50]. For all BKMR models, we ran 50,000 iterations to fit the Markov Chain Monte Carlo (MCMC) sampler. R package *bkmr* [50] was used to obtain point estimates and 95% credible intervals (95% CrI) for BKMR analyses and plots.

#### Definition of relative contributions to EAA

In this study, we defined the term ‘relative contribution’ to reflect the magnitude of importance in the collective association of all six components with EAA. In QGC, the relative contribution is captured by weight, which reflects the proportion of the effect among the individual components with the same direction. In BKMR, the relative contribution is captured by PIP, which reflects the ranked importance of each component in association with EAA. The higher weight and PIP represent the higher importance in the collective association.

#### Sensitivity analyses

To take into account that GrimAge incorporates DNA methylation-based surrogate markers for smoking pack-years [19], we investigated the relative contributions of lifestyle- and health-related components to GrimAA by participant smoking status at Y20. We grouped the participants into ever smokers and never smokers. Additionally, we performed separate analyses by further grouping the participants into current-, former-, and never smokers. We also utilized different measures of physical activity for a sensitivity analysis, using moderate and vigorous activity levels for participants’ physical activity information. We additionally performed stratified analyses by sex and race.

## Supplementary Information

**Additional file 1. Table S1**: Relative contributions of six cumulative lifestyle- and health-related components to PhenoAA in the CARDIA sample from QGC and BKMR. **Table S2**: Relative contributions of six components to PhenoAA in the CARDIA sample from QGC and BKMR, by subgroups. **Table S3**: Estimates from multivariable linear regression models for association between lifestyle- and health-related components and EAA. **Fig. S1**: Spearman correlations among six components by subgroups.

## Data Availability

Data used for this paper were obtained from the Coronary Artery Risk Development in Young Adults (CARDIA) study database (https://www.cardia.dopm.uab.edu). Data can be provided by the corresponding author upon reasonable request.
