# Supplementary material for: Relative contributions of six lifestyle- and health-related exposures to epigenetic aging: the Coronary Artery Risk Development in Young Adults (CARDIA) Study
Source: Clin Epigenetics. 2022 Jul 7;14:85. doi: 10.1186/s13148-022-01304-9 (PMC9264709; doi:10.1186/s13148-022-01304-9)
Supplement: Supplementary file 1 — Additional file 1. Table S1: Relative contributions of six cumulative lifestyle- and health-related components to PhenoAA in the CARDIA sample from QGC and BKMR. Table S2: Relative contributions of six components to PhenoAA in the CARDIA sample from QGC and BKMR, by subgroups. Table S3: Estimates from multivariable linear regression models for association between lifestyle- and health-related components and EAA. Fig. S1: Spearman correlations among six components by subgroups. [file 13148_2022_1304_MOESM1_ESM.docx]

**Supplementary Materials**

Supplementary Table 1. Relative contributions of six cumulative lifestyle- and health-related components to PhenoAA in the CARDIA sample from QGC and BKMR.

Supplementary Table 2. Relative contributions of six components to PhenoAA in the CARDIA sample from QGC and BKMR, by subgroups.

Supplementary Table 3. Estimates from multivariable linear regression models for association between lifestyle- and health-related components and EAA.

Supplementary Figure 1. Spearman correlations among six components by subgroups.

**Supplementary Table 1. Relative contributions of six cumulative lifestyle- and health-related components to PhenoAA in the CARDIA sample from QGC and BKMR.**

| Lifestyle components at or by Y20 | Weights from QGC | PIPs from BKMR |
| --- | --- | --- |
| Alcohol consumption | 0.079 | 0.108 |
| Diet quality | -0.690 | 0.136 |
| Education years | 0.060 | 0.072 |
| Physical activity | -0.233 | 0.120 |
| Sleep hours | -0.077 | 0.096 |
| Smoking | 0.861 | 0.955 |
| *Collective association†* | 1.64 (95% CI=0.15, 3.13), p=0.031 | 1.30 (95% CrI=-0.89, 3.49) |

Models were adjusted for race, sex, body mass index (BMI), and field center; CI: confidence interval; CrI: credible interval.

QGC: Quantile-based g-computation; BKMR: Bayesian kernel machine regression; PIPs: posterior inclusion probabilities. The positive and negative weights from QGC represents the proportion of the effect estimate for each component (sum up to 1 or -1 for the same direction); The PIP reflects the ranked importance of each component in association with PhenoAA.
*†* Change in mean PhenoAA per one quartile change of all six components for QGC; change in mean PhenoAA when all of the six lifestyle components are fixed at their 75th percentile compared to when the six lifestyle components are at their 25th percentile for BKMR.

**Supplementary Table 2. Relative contributions of six components to PhenoAA in the CARDIA sample from QGC and BKMR, by subgroups.**

| Lifestyle components at or by Y20 | Weights from QGC | | PIPs from BKMR | |
| --- | --- | --- | --- | --- |
| By sex | Men  (N=391) | Women  (N=353) | Men  (N=391) | Women  (N=353) |
| Alcohol consumption | 0.106 | 0.282 | 0.184 | 0.121 |
| Diet quality | -0.090 | -0.634 | 0.163 | 0.169 |
| Education years | 0.053 | -0.031 | 0.133 | 0.101 |
| Physical activity | -0.609 | -0.335 | 0.210 | 0.089 |
| Sleep hours | -0.301 | 0.019 | 0.143 | 0.089 |
| Smoking | 0.841 | 0.699 | 0.728 | 0.444 |
| *Collective association* | 1.75 (95% CI=-0.41, 3.92), p=0.114 | 0.65 (95% CI=-1.40, 2.70), p=0.533 | 1.75  (95% CrI=-0.96, 4.47) | 0.68  (95% CrI=-1.79, 3.16) |
| By race | Black participants (N=304) | White participants (N=440) | Black participants (N=304) | White participants (N=440) |
| Alcohol consumption | -0.006 | 0.218 | 0.075 | 0.326 |
| Diet quality | 0.120 | -0.798 | 0.140 | 0.736 |
| Education years | 0.151 | 0.055 | 0.132 | 0.250 |
| Physical activity | -0.994 | -0.051 | 0.177 | 0.232 |
| Sleep hours | 0.044 | -0.151 | 0.048 | 0.285 |
| Smoking | 0.685 | 0.727 | 0.471 | 0.545 |
| *Collective association* | 2.86 (95% CI=0.29, 5.43), p=0.029 | 0.84 (95% CI=-1.01, 2.69), p=0.372 | 2.46  (95% CrI= -0.91, 5.84) | 0.24  (95% CrI= -2.51, 2.99) |

Models were adjusted for race, body mass index (BMI), and field center; CI: confidence interval; CrI: credible interval.

QGC: Quantile-based g-computation; BKMR: Bayesian kernel machine regression; PIPs: posterior inclusion probabilities. The positive and negative weights from QGC represents the proportion of the effect estimate for each component (sum up to 1 or -1 for the same direction); The PIP reflects the ranked importance of each component in association with PhenoAA.
*†* Change in mean PhenoAA per one quartile change of all six components for QGC; change in mean PhenoAA when all of the six lifestyle components are fixed at their 75th percentile compared to when the six lifestyle components are at their 25th percentile for BKMR.

**Supplementary Table 3. Estimates from multivariable linear regression models for association between lifestyle- and health-related components and EAA.**

| Lifestyle components at or by Y20† | All | By sex | | By race | |
| --- | --- | --- | --- | --- | --- |
|  | Total  (N=848) | Men  (N=401) | Women  (N=447) | Black participants (N=346) | White participants (N=502) |
| GrimAA |  |  |  |  |  |
|  | beta^¥^ (95% CI) | beta (95% CI) | beta (95% CI) | beta (95% CI) | beta (95% CI) |
| Alcohol consumption | 0.37 (0.08, 0.66)* | 0.47 (0.05, 0.90)* | 0.26 (-0.12, 0.65) | 0.64 (0.19, 1.10)** | 0.19 (-0.18, 0.56) |
| Diet quality | -0.38 (-0.67, -0.05)* | -0.35 (-0.78, 0.07) | -0.50 (-0.97, -0.03)* | -0.40 (-0.90, 0.09) | -0.47 (-0.88, 0.05) |
| Education years | -0.42 (-0.72, -0.13)** | -0.68 (-1.09, -0.27)** | -0.09 (-0.52, 0.34) | 0.19 (-0.30, 0.69) | -0.88 (-1.25, -0.52)*** |
| Physical activity | 0.10 (-0.21, 0.33) | 0.55 (0.09, 1.01) | -0.13 (-0.55, 0.28) | -0.10 (-0.61, 0.39) | 0.41 (0.03, 0.79)* |
| Sleep hours | 0.06 (-0.21, 0.33) | 0.20 (-0.23, 0.63) | 0.01 (-0.35, 0.36) | 0.01 (-0.36, 0.38) | 0.35 (-0.07, 0.78) |
| Smoking | 1.98 (1.70, 2.27)*** | 2.13 (1.73, 2.54)*** | 1.79 (1.39, 2.19)*** | 2.08 (1.59, 2.58)*** | 1.87 (1.53, 2.21)*** |
| PhenoAA |  |  |  |  |  |
|  | beta^¥^ (95% CI) | beta (95% CI) | beta (95% CI) | beta (95% CI) | beta (95% CI) |
| Alcohol consumption | 0.04 (-0.41, 0.49) | -0.24 (-0.91, 0.43) | 0.27 (-0.32, 0.87) | 0.12 (-0.57, 0.81) | 0.04 (-0.54, 0.64) |
| Diet quality | -0.16 (-0.64, 0.32) | -0.04 (-0.73, 0.64) | -0.32 (-1.03, 0.39) | 0.45 (-0.30, 1.21) | -0.84 (-1.50, 0.18)* |
| Education years | 0.15 (-0.31, 0.61) | -0.02 (-0.67, 0.63) | 0.34 (-0.33, 1.00) | 0.37 (-0.38, 1.14) | 0.06 (-0.52, 0.65) |
| Physical activity | -0.15 (-0.62, 0.32) | -0.04 (-0.78, 0.70) | -0.22 (-0.86, 0.42) | -0.45 (-1.22, 0.32) | 0.20 (-0.41, 0.81) |
| Sleep hours | -0.05 (-0.48, 0.36) | 0.24 (-0.46, 0.94) | -0.27 (-0.81, 0.27) | -0.10 (-0.67, 0.46) | 0.15 (-0.52, 0.83) |
| Smoking | 0.68 (0.24, 1.12)** | 0.94 (0.29, 1.59)** | 0.47 (-0.14, 1.09) | 0.72 (-0.03, 1.48) | 0.58 (0.04, 1.12)* |

Models were adjusted for race, sex, body mass index (BMI), and field center; CI: confidence interval; CrI: credible interval.
* P-value <0.05; ** p-value <0.01; *** p-value <0.001.
† Lifestyle components were standardized to have mean of zero and standard deviation of 1; ¥ The beta coefficients from the linear regression model represents change in GrimAA (unit of year) per one standard deviation increase.

**Supplementary Figure 1. Spearman correlations among six components by subgroups.**


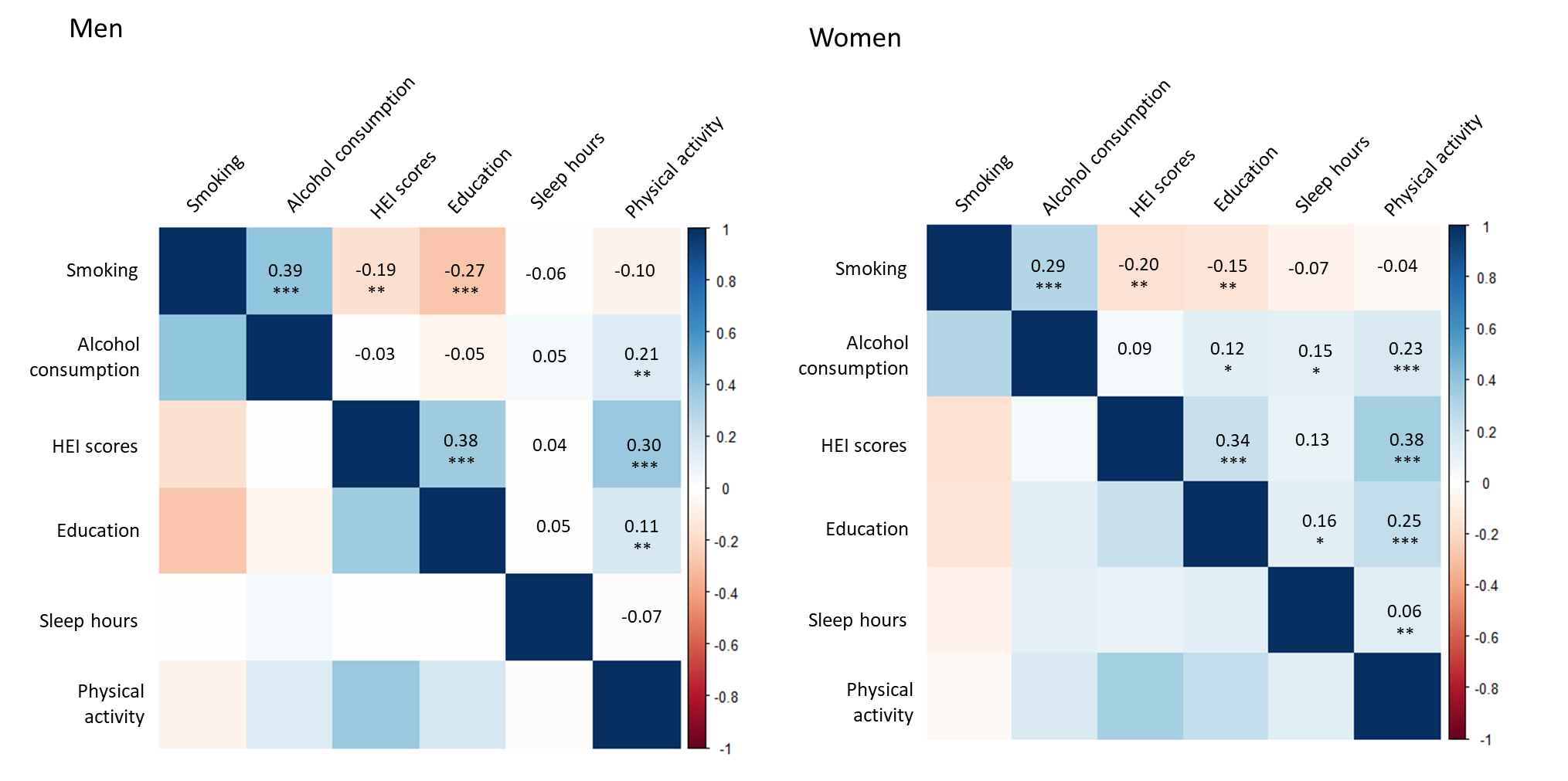

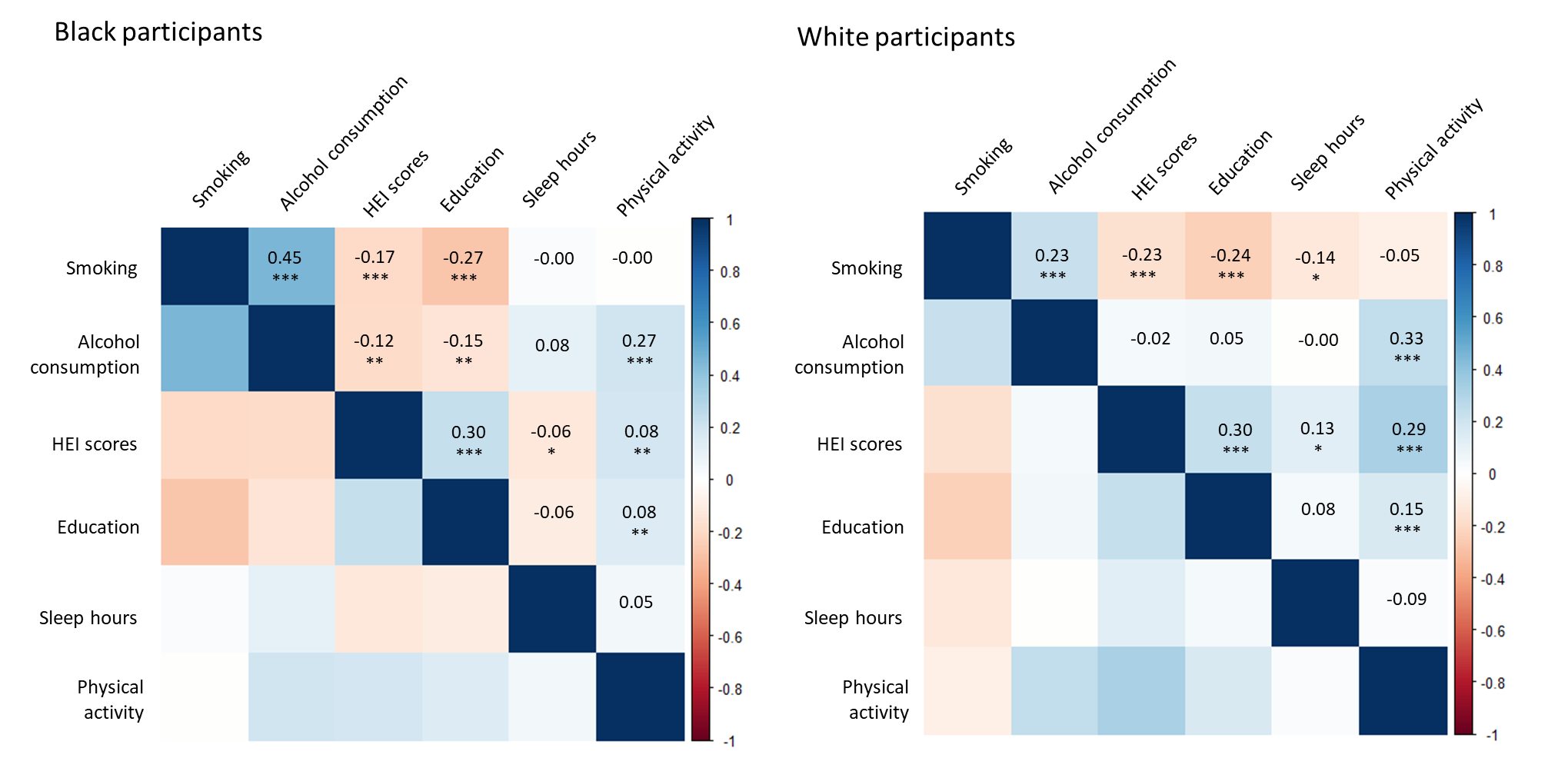


* P-value <0.05; ** p-value <0.01; *** p-value <0.001.
